# Dr. Cornelius Kollock

**Published:** 1897-11

**Authors:** 


Dr. Cornelius Kollock, of Cheraw, South Carolina, died at his home August 16, 1897, aged 73 years. He was graduated from Brown University in 1845, and from the medical department of the University in 1848. After spending two years in Paris he returned to this country and began the practice of his profession, which he pursued with continued and increasing success until his last illness. Dr. Kollock was one of the most prominent physicians in the south and had occupied many professional offices, among which may be mentioned the following: president of the Pee-Dee Medical Society for many years; of the South Carolina Medical Association in 1887; vice-president of the American Gynecological Society in 1892, and president of the Southern Surgical and Gynecological Association in 1894.

Dr. Kollock was widely known and greatly respected for his many qualities of head and heart—virtue, uprightness, ability, faithfulness and good companionship.

